# Evaluating satisfaction with the quality and provision of end-of-life care for patients from diverse ethnocultural backgrounds

**DOI:** 10.1186/s12904-021-00841-z

**Published:** 2021-09-17

**Authors:** Ayah Nayfeh, Christopher J. Yarnell, Craig Dale, Lesley Gotlib Conn, Brigette Hales, Tracey Das Gupta, Anita Chakraborty, Ruxandra Pinto, Ru Taggar, Robert Fowler

**Affiliations:** 1 Institute of Health Policy, Management and Evaluation, Dalla Lana School of Public Health, University of Toronto, 155 College Street, Room 500, Toronto, Ontario M5T 3M7 Canada; 2 Interdepartmental Division of Critical Care Medicine, University of Toronto, Toronto, ON Canada; 3 Sinai, Health Systems, Toronto, ON Canada; 4 Sunnybrook Research Institute, Toronto, ON Canada; 5 Lawrence S. Bloomberg Faculty of Nursing, University of Toronto, Toronto, ON Canada; 6 Sunnybrook Health Sciences Centre, Toronto, ON Canada; 7 H. Barrie Fairley Professorship of Critical Care at the University Health Network, Toronto, ON Canada

**Keywords:** End-of-Life Care, Satisfaction, Quality of Care, Race/Ethnicity, Culture

## Abstract

**Background:**

Recently immigrated and ethnic minority patients in Ontario, Canada are more likely to receive aggressive life-prolonging treatment at the end of life in comparison to other patients. To explore this finding further, this survey-based observational study aimed to evaluate satisfaction with the quality of end-of-life care for patients from diverse ethnocultural backgrounds.

**Methods:**

The End-of-Life Satisfaction Survey was used to measure satisfaction with the quality of inpatient end-of-life care from the perspective of next-of-kin of recently deceased patients at Sunnybrook Health Sciences Centre in Toronto, Ontario (between March 2012 to May 2019). The primary outcome was the global rating of satisfaction. Associations with patient ethnicity, patient religion, level of religiosity/spirituality, language/communication barriers, and location of death were assessed using univariable and multivariable modified Poisson regression. Secondary outcomes included differences in satisfaction and rates of dying in intensive care units (ICU) among patient population subgroups, and identification of high priority areas for quality-of-care improvement.

**Results:**

There were 1,543 respondents. Patient ethnicities included Caucasian (68.2%), Mediterranean (10.5%), East Asian (7.6%), South Asian (3.5%), Southeast Asian (2.1%) and Middle Eastern (2.0%); religious affiliations included Christianity (66.6%), Judaism (12.3%) and Islam (2.1%), among others. Location of death was most commonly in ICU (38.4%), hospital wards (37.0%) or long-term care (20.0%). The mean(SD) rating of satisfaction score was 8.30(2.09) of 10. After adjusting for other covariates, satisfaction with quality of end-of-life care was higher among patients dying in ICU versus other locations (relative risk [RR] 1.51, 95%CI 1.05-2.19, p=0.028), lower among those who experienced language/communication barriers (RR 0.49 95%CI 0.23-1.06, p=0.069), and lower for Muslim patients versus other religious affiliations (RR 0.46, 95%CI 0.21-1.02, p=0.056). Survey items identified as highest priority areas for quality-of-care improvement included communication and information giving; illness management; and healthcare provider characteristics such as emotional support, doctor availability and time spent with patient/family.

**Conclusion:**

Satisfaction with quality-of-care at the end of life was higher among patients dying in ICU and lower among Muslim patients or when there were communication barriers between families and healthcare providers. These findings highlight the importance of measuring and improving end-of-life care across the ethnocultural spectrum.

**Supplementary Information:**

The online version contains supplementary material available at 10.1186/s12904-021-00841-z.

## Background

Racial and ethnic differences in care delivery and outcomes at the end of life are well-documented [1]. Although a majority of Canadians have expressed preferences to die at home [2], prior studies have shown a strong association between patient race/ethnicity with increased use of life-prolonging treatments and admission to intensive care units (ICU) at the end of life [3–7]. Among decedents in Ontario, recently immigrated and ethnic minority patients were significantly more likely to die in the ICU and more likely to receive aggressive life-prolonging treatment in the last six months of life in comparison to other patients [6].

It is not clear whether the observed variation in healthcare at end of life according to race/ethnicity or immigration status is attributable to individual or ethnocultural preferences for end-of-life care [4, 5, 8]; or, whether this is a result of specific disparities in the quality of care – such as communication barriers – that may occur along the end-of-life trajectory [9, 10]. Demonstrating the distinction between preference-driven differences and healthcare disparities is important for improving the quality and equity of end-of-life care [11]. Prior studies have identified priorities for improving the quality of care for patients with serious illness, such as avoiding unwanted use of life-prolonging treatment, effective communication with the healthcare team, and having trust and confidence in clinicians providing care [12–16]; however, these studies represented mostly Caucasian patients or reported on experiences within the US healthcare system.

The multicultural Canadian population provides an opportunity to gain insight into the end-of-life care experiences of patients from diverse ethnocultural backgrounds. This study aimed to evaluate satisfaction with the quality of end-of-life care for patients of diverse ethnocultural backgrounds from the perspective of bereaved family members. The primary aim was to evaluate the association between satisfaction with quality of end-of-life care and patient ethnocultural characteristics. The secondary aim was to identify high priority areas for quality-of-care improvement. We hypothesized that satisfaction with quality of end-of-life care would be lower for family members of patients from non-Caucasian, non-Christian backgrounds in comparison to other patients.

## Methods

### Study design

This observational survey-based study used the End-of-Life Satisfaction survey to measure family satisfaction with inpatient end-of-life care in a large academic tertiary care Canadian hospital. The End-of-Life Satisfaction Survey is a validated 52-item tool that was modified from the National Research Corporation (NRC) Hospice Survey [17, 18] to include patient demographic items that allowed for examination of their respective associations with the global rating of satisfaction (Additional file 1). These items included: patient race/ethnicity (Caucasian, Caucasian-Mediterranean, Black, East Asian, South Asian, Southeast Asian, Middle Eastern, Hispanic, First Nations, and other); patient religion [Atheist, Buddhist, Christian (all denominations), Hindu, Jehovah’s Witness, Jewish, Mormon, Muslim, Sikh, no religion, other]; level of religiosity/spirituality; and preferred spoken language. A variety of Likert scales were used to measure satisfaction (5-point Likert scale: 1-very dissatisfied, 2-dissatisfied, 3-neither, 4-satisfied, 5-very satisfied) and presence of care (5-point Likert scale: 1-never, 2-sometimes, 3-usually, 4-always, 5-do not know) around key domains of inpatient care (i.e., coordination of care, physical comfort and emotional support, involvement of family/friends, respect for patient preferences, and overall impression). The overall global rating of satisfaction was measured using a 10-point Likert scale (where 0=worst care possible and 10=best care possible). Items in the End-of-Life Satisfaction Survey have been shown to have a high degree of face validity, construct validity and internal reliability consistency, which demonstrate its utility for measuring satisfaction with inpatient end-of-life care [18].

### Outcomes

The outcome measure for the primary analysis was the global rating of satisfaction score which asked: *On a scale of 0 to 10 (where 0 means the worst care possible and 10 means the best care possible), what number would you give the overall care that your family member received in the time leading up to their death?* Data points were visually assessed for a natural dividing point via a histogram and scatterplot, leading to subsequent dichotomized levels of satisfaction as “*higher satisfaction”* with the quality of end-of-life care (ratings 6 to 10) and “*lower satisfaction”* with the quality of end-of-life care (ratings 0 to 5). Secondary outcomes focused on rates of dying in the ICU and identification of high priority areas for quality-of-care improvement.

### Definitions

Healthcare literature suggests that discerning the role of race and ethnicity in health outcomes is difficult [19, 20]. *Race* is defined as “any one of the groups that humans are often divided into based on physical traits regarded as common among people of shared ancestry” [21], whereas *ethnicity* is defined as “shared culture, such as language, ancestry, practices, and beliefs” [22]. In accordance with current recommendations [19, 20], the End-of-Life Satisfaction Survey employed a single mutually exclusive term *“race/ethnicity”* to understand factors contributing to racial/ethnic differences in health and elucidate the multidimensional nature of this construct.

### Sampling and data collection

Next-of-kin (hereinafter referred to as family members) for patients who died at Sunnybrook Health Sciences Centre – a large urban hospital in Toronto, Canada with 1,325 beds and more than 33,000 acute care admissions annually – were routinely sent a voluntary and non-incentivized End-of-Life Satisfaction Survey, which was administered by post-mail through NRC Health Canada. Survey respondents were recruited between March 2012 to May 2019. Previous work has shown that close patient proxies can reliably report on the quality and provision of end-of-life care on behalf of dying patients given their close knowledge and experience during the patient’s last weeks of life [23]. Although previous research suggests that there is a low likelihood of distress among family members participating in end-of-life care survey research [24], next-of-kin of deceased patients were sent the End-of-life Satisfaction Survey at least four to six weeks after death to reduce the potential of any distress in receiving a survey so close to the patient’s death. The four-to-six week timeframe was informed by previous survey-based studies evaluating satisfaction at the end of life [15, 16].

The invitation letter accompanying the survey explained the confidential and voluntary nature of the request. One reminder survey was sent three weeks after the initial mail-out to those who did not respond. The mail-out excluded families actively involved in institutional or medical-legal claims as identified by the Sunnybrook Patient Experience Office; patients who died in maternal, newborn or neonatal units, or in the emergency department *prior* to hospital admission; and patients who did not have next-of-kin listed in the electronic records. Anonymized responses were returned by post-mail. Family member responses were not linked to individual hospital-based patient records after the initial mail-out request. Thus, a respondent vs. non-respondent analysis could not be performed.

### Characteristics of patients

Patient characteristics and demographics included race/ethnicity, religion, level of religiosity/spirituality, preferred spoken language and language barriers (as reported by family respondents).The database also reported on patient location of death. Information on patient sex, socioeconomic status, cause of death, or goals of care nearing the end of life (e.g., choice for cardiopulmonary resuscitation) was not reported in the database. However, ratings of satisfaction reflect family-reported experience of outcomes of care and the healthcare delivered to patients.

### Data analysis

#### Statistical analysis

The normality of distribution for all responses were assessed both numerically and visually to determine appropriate parametric or non-parametric analyses. Modified Poisson regression was used to analyze binary data for relative risk (RR) with robust error variance [25]. A missing value analysis was also performed on the set of returned surveys to ensure that missing values were not correlated to other covariates or the outcome [26].

#### Power analysis

This study used an existing dataset with a fixed sample size (n=1,543). However, *a priori*, to inform our choice of primary and secondary analyses, we conducted a power analysis to determine the minimal detectable effect that could be found in the global end-of-life care satisfaction question among respondents who identified patient race/ethnicity (n=1,384) [27]. Using α=0.05 and a desired power of 0.8, we found that a small effect size of 0.18 in relative risk ratio could be detected between two independent samples (Additional file 1).

#### Primary analysis

Potential explanatory variables for satisfaction with the quality of end-of-life care were selected *a priori* according to variables available in the dataset, clinical experience, and a directed acyclic graph based on clinical relevance to the research question and prior research [3–5, 13, 16, 28, 29]. These variables included: 1) patient race/ethnicity, 2) patient religion, 3) level of religiosity/spirituality, 4) language/communication barriers and 5) location of death. These five variables were used to build a multivariable Poisson regression model to explore how patient ethnicity and/or religion, in addition to the other explanatory variables, influenced satisfaction with the quality of end-of-life care.

To begin, univariable Poisson regression was used to explore the association between the global rating of satisfaction and each candidate predictor variable. To examine associations with patient race/ethnicity and religion with satisfaction, separate stratified analyses were performed with each patient ethnic or religious group to identify important differences in ratings of satisfaction [at p-value of <0.2 [30]] and to inform potential groupings of certain geographically or culturally-similar ethnic groups. The Caucasian group included patients who were identified as Caucasian or Caucasian-Mediterranean (e.g., Italian, Greek, Spanish). We did not assess specific patient ethnic or religious groups with low cell counts (fewer than five respondents); however, these patients were included in the reference category.

The other explanatory variables were dichotomized into the following categories for analyses: level of religiosity/spirituality (*very/somewhat religious* vs. *not at all/a little religious*), language barriers (*usually/always* vs. *never/sometimes*) and location of death (*ICU* vs. *other*). The ICU setting was selected to explore the potential influence of dying in the setting of critical illness and organ-support, but with a high nurse-to-patient ratio, relative to patients who died in hospital wards, short-stay units, long-term care (including palliative care) or the emergency department. We did not include *“I don’t know”* responses in the analysis after confirming that there were no significant differences in ratings of satisfaction between those who experienced/had an opinion around specific survey items and those who did not know.

All variables that showed important potential differences in satisfaction with the quality of end-of-life care (p-value of <0.2) in the univariable analyses were entered into a multivariable Poisson regression model using the global rating of satisfaction score as the dichotomized outcome. We measured the probability of being *less satisfied* with the quality of end-of-life care. Results were presented in the reciprocal form to help with interpretation, such that relative risk ratios greater than one (>1) represent higher ratings of satisfaction and relative risk ratios less than one (<1) represent lower ratings of satisfaction. We tested for multicollinearity using Pearson’s chi-square test and variance inflation factors and removed variables that were highly correlated or predictive of one another. A sensitivity analysis was performed to assess the potential impact on results of using different binary cut-off points for the satisfaction score.

#### Secondary analyses: Importance-satisfaction grid

Areas of high-priority for quality improvement were identified through an Importance-Satisfaction grid, which plotted the percent of satisfied responses for each survey item by their Spearman correlation coefficient with the global rating of satisfaction (at p-value of <0.05) [13, 16]. Four quadrants were established by plotting vertical and horizontal lines at the median values. Survey items considered highest priority for improvement were those most strongly correlated with the global rating of satisfaction yet had a lower proportion of satisfied responses.

Results are described in terms of relative risk (RR – the ratio of proportions of a specific group compared with all other groups) with a significance threshold level of α=0.05. Descriptive statistics included counts and proportions for categorical variables and means (standard deviations) and medians (interquartile ranges) for continuous variables. Statistical analysis was conducted using IBM SPSS Statistics for Macintosh, Version 26.0 (IBM Corp., Armonk, N.Y., USA) [31]. All methods were carried out in accordance with relevant guidelines and regulations.

## Results

The survey dataset included 1,543 responses from March 2012 to May 2019. The response rate was 37.7%. Respondents were most commonly the partner/husband/wife of the patient (n=723, 48.7%), followed by son/daughter (n=360, 24.3%). The most common location of death for patients was the ICU (n=592, 38.4%), hospital ward (n=571, 37.0%) and long-term care units (n=309, 20.0%) (Table 1). Overall, the mean rating of satisfaction score was skewed towards the higher end of the 10-point Likert scale (mean=8.30, median=9.00, SD=2.09). Histograms, normal Q-Q plots and boxplots confirmed a non-normal or non-parametric data distribution.

**Table 1:** Characteristics of patients and family member respondents

	N (%)
Patient Ethnicity	
Caucasian	944 (68.2)
Caucasian-Mediterranean (Italian, Greek, Spanish)	145 (10.5)
East Asian (Chinese, Japanese, Korean)	105 (7.6)
South Asian (East Indian, Pakistani, Sri Lankan)	49 (3.5)
Southeast Asian (Vietnamese, Filipino, Indonesian)	29 (2.1)
Middle Eastern (Persian, Turkish, Lebanese, Egyptian)	28 (2.0)
Black	25 (1.8)
First Nations	5 (0.4)
Hispanic	5 (0.4)
Other	49 (3.5)
Patient Religion	
Christian	936 (66.6)
Jewish	173 (12.3)
No religion - but spiritual	130 (9.2)
Atheist	49 (3.5)
Muslim	29 (2.1)
Buddhist	19 (1.4)
Hindu	16 (1.1)
Jehovah’s Witness	6 (0.4)
Sikh	2 (0.1)
Other	45 (3.2)
Patient Preferred Spoken Language	
English	1112 (81.9)
Italian	58 (4.3)
Chinese	56 (4.1)
Russian	13 (1.0)
Other	118 (8.7)
Family Preferred Spoken Language	
English	1260 (93.8)
Chinese	29 (2.2)
Other	54 (4.0)
Relationship to Patient	
Husband/Wife/Partner	723 (48.7)
Son/Daughter	360 (24.3)
Parent	222 (15.0)
Brother/Sister	70 (4.7)
Friend	34 (2.3)
Son-in-Law/Daughter-in-Law	14 (0.9)
Other	61 (4.1)
Location of Death	
Intensive Care Units	592 (38.4)
Hospital Ward	571 (37.0)
Long-Term Care	309 (20.0)
Emergency Room	53 (3.4)
Other	18 (1.2)
Language Barriers	
Usually/always	36 (2.4)
Never/sometimes	1461 (97.6)
Level of Religiosity/Spirituality	
Very/somewhat	680 (51.6)
A little/not at all	637 (48.4)

Among 1,384 respondents who completed the patient demographic items, 295 (21.3%) patients were identified with a racial/ethnic group that was not Caucasian (Table 1). A missing value analysis indicated that missing values in the set of returned surveys were missing completely at random (Little’s MCAR test, p=0.1).

### Satisfaction with end-of-life care

Univariable analyses of patient race/ethnicity, religion, religiosity, language/communication barriers and location of death identified factors for inclusion in the multivariable model (Table 2). The mean global ratings of satisfaction by patient race/ethnicity and patient religion are presented in Figures A1 and A2 in Additional file 1. There were no significant differences in rates of dying in ICU among patient racial/ethnic or religious subgroups. Due to high collinearity between Middle Eastern and Muslim patient populations (*X*^*2*^(1)=200.73, p<0.001, Cramer’s V=0.387), both variables could not reasonably be included in the final model, and Muslim (vs. not Muslim) was preferentially selected for entry into the multivariable model on the basis of greater variable specificity and significance level in the univariable analyses.

**Table 2:** Patient Demographic and Care Delivery Characteristics Associated with the Global Rating of Satisfaction

Variables	Univariable Poisson Regression		Multivariable Poisson Regression	
	Unadjusted Relative Risk (RR) CI (95%)	p-value	Adjusted Relative Risk (RR) CI (95%)	p-value
Patient Race/Ethnicity				
Caucasian vs. not Caucasian	1.29 (0.89-1.87)	0.172	1.11 (0.73-1.70)	0.617
South Asian vs. not South Asian	1.21 (0.47-3.14)	0.696	-	-
East Asian vs. not East Asian	1.12 (0.59-2.14)	0.730	-	-
Southeast Asian vs. not Southeast Asian	0.95 (0.32-2.80)	0.921	-	-
Black vs. not Black	0.75 (0.26-2.18)	0.593	-	-
Middle Eastern vs. not Middle Eastern	0.52 (0.23-1.17)	0.113	-	-
Other Race/Ethnicity vs. all other groups	0.48 (0.26-0.89)	0.019	-	-
Patient Religion				
Buddhist vs. not Buddhist	1.82 (0.27-12.32)	0.539	-	-
Christian vs. not Christian	1.22 (0.88-1.70)	0.225	-	-
Jewish vs. not Jewish	1.12 (0.67-1.87)	0.661	-	-
Atheist vs. not Atheist	0.99 (0.42-2.30)	0.972	-	-
No religion vs. all other religious groups	0.83 (0.50-1.37)	0.466	-	-
Hindu vs. not Hindu	0.80 (0.22-2.96)	0.741	-	-
Muslim vs. not Muslim	0.46 (0.22-0.95)	0.036	0.46 (0.21-1.02)	0.056
Other Religion vs. all other groups	0.61 (0.30-1.22)	0.158	-	-
Patient Religiosity				
Very/somewhat vs. a little/not at all	1.15 (0.83-1.59)	0.412	-	-
Language/Communication Barriers				
Usually/always vs. never/sometimes	0.57 (0.27-1.20)	0.137	0.49 (0.23–1.06)	0.069
Location of Death				
ICU vs. other	1.34 (0.97-1.85)	0.081	1.51 (1.05–2.19)	0.028

In the multivariable analyses, after adjusting for patient race/ethnicity, location of death and language/communication barriers, family members of patients who died in ICU had greater satisfaction with the quality of end-of-life care in comparison to patients who died in other units (RR 1.51, 95% CI 1.05-2.19, p=0.028). Family members of Muslim patients were less satisfied with the quality of end-of-life care in comparison to other religious affiliations (RR 0.46, 95% CI 0.21-1.02, p=0.056). A multivariable regression analysis using a multi-categorical variable combining patient ethnicity and religion had similar findings (Table A1 in Additional file 1). Those who experienced language or communication barriers appeared to have lower ratings of satisfaction, although this finding was not statistically significant (RR 0.49 95%CI 0.23–1.06, p=0.069) (Table 2).

### Priority areas for quality improvement

**Fig. 1:**
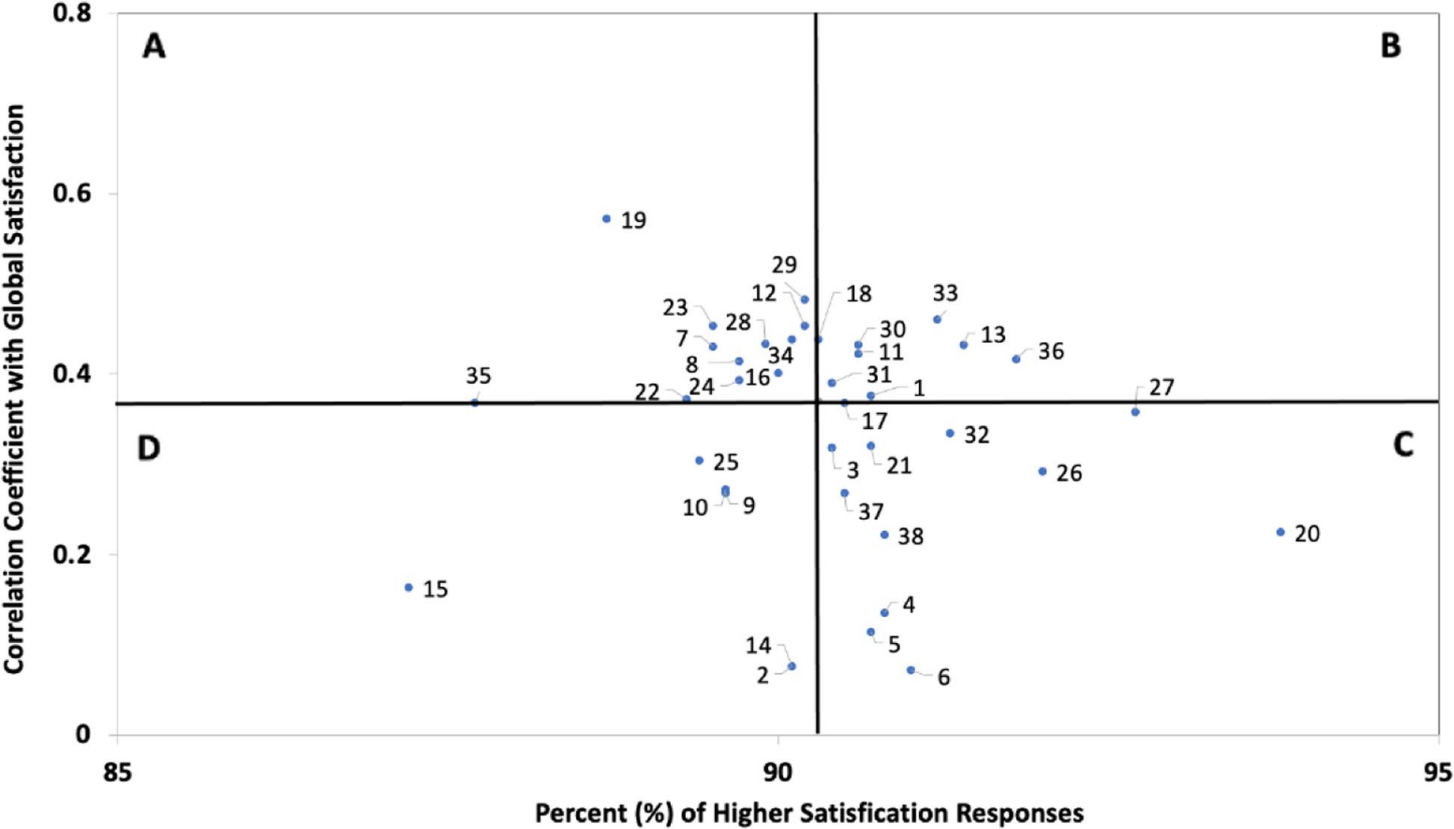
Importance-Satisfaction Grid of High Priority Areas for Quality Improvement. ^a^ Importance-Satisfaction grid represents family member responses on behalf of all patient decedents (n=1477). ^b^ Correlation coefficients for survey items were calculated using Spearman’s Rho and significant coefficients are represented on the grid (p<0.05). ^c^ The median correlation coefficient was r_s_=0.368 and 90.3% for median percent of higher satisfaction responses. ^d^ Survey items represented above include: Care was consistent with patient wishes (1); Language was communication barrier (2); Religious and spiritual needs addressed (3); Problem understanding doctor regarding what to expect (4); Problem understanding nurse regarding what to expect (5); Problem understanding social worker regarding what to expect (6); Doctor listened to concerns (7); Nurse listened to concerns (8); Social worker listened to concerns (9); Chaplain listened to concerns (10); Amount of information doctors provided (11); Kept informed about family members condition (12); Satisfied that you received consistent information (13); Family member had specific wishes re: treatment (14); Discussed Living Will/POA with health care team (15); Told how pain would be treated (16); Amount of help family member got with breathing (17); Received info on meds for pain/shortness of breath (18); Amount of help dealing with anxiety/sadness (19); Pain well-controlled (20); Family member treated with respect/dignity (21); Concerns about personal care needs being met (22); Wanted more info on what to expect while dying (23); Amount of support at time of family members death (24); Amount of support following family members death (25); Felt supported at the time of death (26); Felt supported after death (27); Able to access doctor when needed (28); Doctor spent enough time with you (29); Confidence/trust in doctor (30); Confidence/trust in nurse (31); Satisfied that doctor explained things honestly (32); Satisfied that you received timely updates (33); Clear which doctor was in charge of care (34); Clear which nurse was in charge of care (35); Satisfied that health care staff worked as a team (36); Problems with doctors not knowing medical history (37); Decisions made without enough family input (38)

**Fig. 2:**
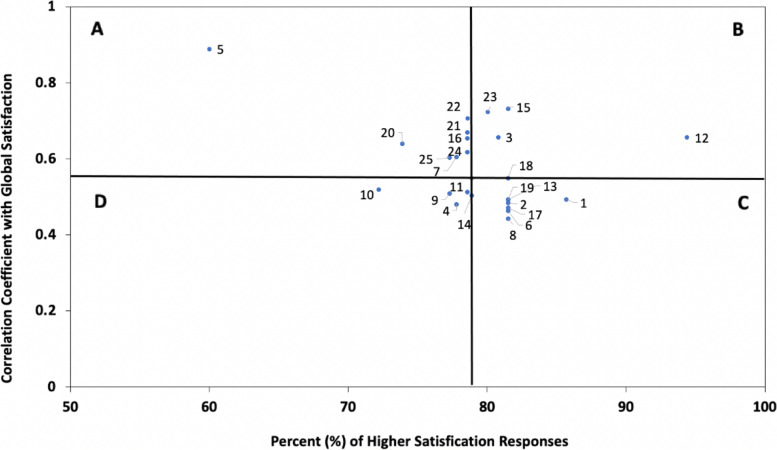
Importance-Satisfaction Grid of High Priority Areas for Quality Improvement for the Muslim Patient Population. ^a^ Importance-Satisfaction grid represents family member responses on behalf of the Muslim patient population (n=28).^b^ Correlation coefficients for survey items were calculated using Spearman’s Rho and significant coefficients are represented on the grid (p<0.05).^c^ The median correlation coefficient was r_s_=0.549 and 78.9% for median percent of higher satisfaction responses.^d^ Survey items represented above include: Care was consistent with patient wishes (1); Decisions made without enough family input (2); Doctor listened to concerns (3); Nurse listened to concerns (4); Chaplain listened to concerns (5); Amount of information doctors provided (6); Kept informed about family members condition (7); Satisfied that you received consistent information (8); Told how pain would be treated (9); Amount of help family member got with breathing (10); Would have liked more info on medications (11); Amount of help dealing with anxiety/sadness (12); Family member treated with respect/dignity (13); Concerns about personal care needs being met (14); Wanted more info on what to expect while dying (15); Able to access doctor when needed (16); Doctor spent enough time with you (17); Confidence/trust in doctor (18); Confidence/trust in nurse (19); Satisfied that doctor explained things honestly (20); Satisfied that you received timely updates (21); Clear which doctor was in charge of care (22); Clear which nurse was in charge of care (23); Satisfied that health care staff worked as a team (24); Problems with doctors not knowing medical history (25)

Importance-Satisfaction plots identified the following survey items as highest priority areas in need of quality improvement among the broader patient population (quadrant A): “doctor listened to concerns [7]”, “nurse listened to concerns [8]”, “kept informed about family members condition [12]”, “told how pain would be treated” [16], “received information on medications for pain/shortness of breath” [18], “amount of help dealing with anxiety/sadness” [19], “concerns about personal care needs being met” [22], “wanted more information on what to expect while dying” [23], “amount of support at time of family members death” [24], “able to access doctor when needed” [28], “doctor spent enough time with you” [29], “clear which doctor was in charge of care” [34] and “clear which nurse was in charge of care” [35] (Fig. 1). Survey items in quadrant D (bottom left) were less correlated with the global rating of satisfaction but were considered potentially worthwhile targets for improvement given the lower proportion of those satisfied with current practice. Among the Muslim patient population, the following survey items were identified as highest priority areas in need of quality improvement (quadrant A): “chaplain listened to concerns [5]”, “kept informed about family members condition” [7], “able to access doctor when needed” [16], “satisfied that doctor explained things honestly” [20], “satisfied with timely updates” [21], “clear which doctor was in charge of care” [22], “satisfied that health care staff worked as a team” [24] and “problems with doctors not knowing medical history” [25] (Fig. 2). High priority areas for quality improvement are presented in Tables A2 and A3 in Additional file 1.

### Sensitivity analysis

A sensitivity analysis was performed on the Muslim patient population to assess the potential impact on results of using different binary cut-off points for the global rating of satisfaction score (Table A4 in Additional file 1). Similar effect sizes were observed across the different binary cut-off values. The use of an ordinal or trichotomized outcome variable detected a smaller but significant effect.

## Discussion

We found that family members of Muslim patients in this study were less satisfied with the quality of inpatient end-of-life care in comparison to patients from other religious backgrounds. Lower ratings of satisfaction persisted after adjusting for patient ethnicity, location of death and language/communication barriers. Contrary to prior evidence [29], we found that family members of patients who died in the ICU were more satisfied with the quality of end-of-life care in comparison to patients who died in other units. We did not find any significant differences in rates of dying in ICU among patient ethnic or religious subgroups.

When examining perceptions around quality of end-of-life care, there are varying expectations for care (such as the appropriate amount of life-prolonging treatment for a dying patient) that could have a mitigating effect on ratings of satisfaction. Lower ratings of satisfaction among specific population subgroups may be influenced by individual and cultural expectations for care based on ethnic origin and/or region of birth. In a large observational cohort study examining end-of-life practices in 730 ICUs across 84 countries, researchers found that decisions to withhold or withdraw life-sustaining treatment were less frequently adopted in South Asia [10% (6-17%)] and the Middle East [18% (12-26%)] and most frequently adopted in Oceania [67% (54-78%)] and North America [65% (55-74%)] [32]. This finding is consistent with population-based studies that also showed that recently immigrated patients from Asia (including the Middle East) [6] and patients of South Asian descent [7] were more likely to die in ICU and received more aggressive end-of-life care (i.e., mechanical ventilation, dialysis, artificial nutrition, etc.) in the last six months of life in comparison to other patients in Ontario, Canada. Differences in ratings of satisfaction may therefore reflect, in part, varying individual and cultural preferences for end-of-life care [3–5].

A large majority of Muslims in Canada are of Asian descent (including the Middle East) and more than a half of the foreign-born Muslim population arrived after the year 2000 (with 37 percent arriving between the years 2001 and 2006) [33]. This might indicate that a significant proportion of Muslims in Canada are more recent immigrants who may be in the process of adjusting and adapting to sometimes different culture and practices within a Canadian healthcare system. Our survey-based analysis, however, did not collect immigration status and we could not separate this potential influence in the multivariable model.

Another key limitation of this study was that the survey was available in English only. This may have limited accessibility of the survey to recent immigrants and/or ethnic minorities who do not speak or read the English language. The survey also did not include culturally specific questions around aspects of care – such as communication preferences, meanings of suffering, and decision-making processes – that may have influenced perceptions around the quality of end-of-life care from the perspective of individuals from diverse ethnocultural backgrounds [34]. For instance, cultural preferences for family decision-making may not have been adequately addressed by healthcare providers in Canada, who tend to place a greater emphasis on patient autonomy and an individual’s right to make decisions around their own healthcare [35]. In-depth qualitative research with specific patient population subgroups, particularly those from Muslim religious backgrounds, can help identify culturally specific questions and factors that influence the quality and provision of end-of-life care to inform future studies. Further qualitative insight can also help elucidate the influence and role of patient gender or sex on the quality and provision of care, which was not collected or explored in this study. This is particularly important as research suggests there are ethnocultural differences in preferences for communication and end-of-life care among Muslim patients and Americans of Arab descent that are compounded by patient sex and gendered roles [36, 37].

Although we were unable to determine through this survey-based analysis whether differences in satisfaction around end-of-life care received result from an inconsistency between expectations of care and the actual care provided, we were able to identify specific aspects of end-of-life care that may have contributed to lower ratings of satisfaction among family members of Muslim patients. High priority areas identified for potential improvement include communication and information-giving, characteristics of healthcare providers, and chaplain support. These survey items were not specific to the Muslim patient population, and target areas reflect a need to connect on typical elements of end-of-life care among the general patient population as well.

High priority areas identified for quality improvement in this study are consistent with prior literature that identified effective communication and access to timely information as key modifiable issues and important measures for improving the quality of end-of-life care among the general patient population [12, 13, 28, 38–40]. In a multi-centre study examining priorities for improving end-of-life care in Canada, aspects of care that were identified as high priority included emotional support provided to patients, the quality of the relationship between doctor, patient and patient’s family, and communication and decision-making [9, 13]. It is not yet clear whether interventions exist to support patients and families in this endeavour, and whether these mechanisms are perceived as acceptable and/or effective by individuals from different ethnocultural backgrounds.

Contrary to some prior evidence [29], our study found that family members of patients who died in ICU were more satisfied with the quality of end-of-life care in comparison to patients who died in other units. This finding is consistent with other studies that suggest that family members of ICU decedents are generally more satisfied with the quality and experience of care as a result of more frequent interaction and engagement with healthcare staff over the course of the patient’s stay in the ICU [41]. Low satisfaction scores at the end of life, on the other hand, have been shown to be strongly correlated with psychological burden and symptoms of anxiety, depression, post-traumatic stress disorder and complicated grief [42]. Opportunities likely exist to enhance emotional support for patients and families moving along the end-of-life trajectory while in hospital.

Quality measures focusing on emotional/spiritual support and effective and timely communication with patients and families have been previously proposed as specific targets for improving the quality of care at the end of life [38]. For example, many hospitals settings have chaplains (“spiritual care practitioners”) available on-site to provide spiritual care to dying patients and their families and to accommodate religious needs. While some hospitals may be able to provide more consistent access to Protestant, Catholic and Jewish religious leaders, due to the relative proportion of religions among patient populations, absence of community leaders and supports for individuals of less common faiths in our hospital setting – for example, Islamic faith in this study – may have influenced our findings. Efforts to improve access to diverse community and religious leaders for patients and families from different ethnocultural backgrounds might lead to better clinical outcomes and improved satisfaction at the end of life.

Findings from this study underscore the need to identify and remedy disparities in care delivery and/or expectations for care that may be contributing to lower ratings of satisfaction at the end of life. Insight into factors leading to variation in satisfaction with the quality of end-of-life care can improve the delivery of high quality and equitable care, particularly in healthcare settings with diversity in culture, religion and ethnicity. Associations emerging from this study inform, but do not resolve, the question of whether preferences, disparities or a combination of these or other factors, account for family satisfaction or dissatisfaction with the quality of end-of-life care. Further complementary quantitative and qualitative research designs are important for more fully characterizing hypothesized relationships and understanding culturally unique needs at the end of life.

### Limitations

Observational survey-based study designs are limited in that identified associations could have multiple explanations, and it can be difficult to derive temporality in potential relationships using a one-time measurement [43]. For example, we are not able to determine whether dissatisfaction causes emotional distress, or whether emotional distress causes dissatisfaction with care. The minimal important difference (MID) for patient satisfaction surveys is also not known, and it is difficult to interpret whether differences in satisfaction scores are, in fact, meaningful or clinically important to patients/families [44]. Efforts were made to control for known and measured confounding variables using adjusted regression models at the analysis stage. However, the survey did not collect the length of time or exposure to inpatient care at Sunnybrook Health Sciences Centre, and we could not adjust for decedents’ age, sex, income or disease category.

The voluntary sampling approach may introduce a respondent selection bias, as people who tend to respond to surveys often have stronger opinions compared to non-responders [45]. There is also a potential for recall bias given the retrospective nature of this survey. To minimize both selection and recall bias, the survey was mailed out each month to family members who had a loved one die at Sunnybrook Health Sciences Centre (at least four to six weeks after death). However, the survey was only sent to family members of patients who had a next-of-kin listed in their electronic records, and there is the potential that decedents without a next-of-kin listed could have a different end of life experience, especially if this represented a different degree of family support or presence while in the hospital. This study was also limited to patients who died at Sunnybrook Health Sciences Centre and findings may not be representative of patients who died in other settings outside of the hospital (i.e., home-based palliative care). The survey is also available in English only and may have been less accessible to recent immigrants and/or ethnic minorities who do not speak or read the English language. Surveys were not linked to individual hospital-based patient records after the initial mail-out request and we could not perform a missing value analysis to assess whether the returned surveys were missing fully at random.

## Conclusion

In this study, satisfaction with the quality of end-of-life care was higher among patients dying in ICU compared to other hospital units and lower among Muslim patients or when there were language/communication barriers between patients, families and the healthcare team. These findings underscore the need to identify and remedy potential disparities in care delivery and/or ethnocultural expectations for care that may contribute to lower satisfaction at the end of life. Complementary qualitative research designs should seek to capture – more explicitly – the experiences of patients and families from specific ethnocultural backgrounds. More insight to the patient care experience and decision-making process at the end of life will further our understanding of factors that constitute quality care and culturally unique gaps that exist along the end-of-life trajectory.

## Supplementary Information

**Additional file 1.**

## Data Availability

The data that support the findings of this study are available from Sunnybrook Health Sciences Centre, but restrictions apply to the availability of these data, which were used under license for the current study, and so are not publicly available. Data are however available from the authors upon reasonable request and with permission of Sunnybrook Health Sciences Centre.

## Abbreviations

ICU: Intensive Care Unit
MID: Minimal Important Difference
NRC: National Research Corporation
REB: Research Ethics Board
RR: Relative Risk
