# Supplementary material for: Evaluating satisfaction with the quality and provision of end-of-life care for patients from diverse ethnocultural backgrounds
Source: BMC Palliat Care. 2021 Sep 17;20:145. doi: 10.1186/s12904-021-00841-z (PMC8449427; doi:10.1186/s12904-021-00841-z)
Supplement: Supplementary file 1 — Additional file 1. [file 12904_2021_841_MOESM1_ESM.docx]

## Appendix A

**Figure A1 – Boxplot of Global Rating of Satisfaction Scores by Patient Race/Ethnicity**

**
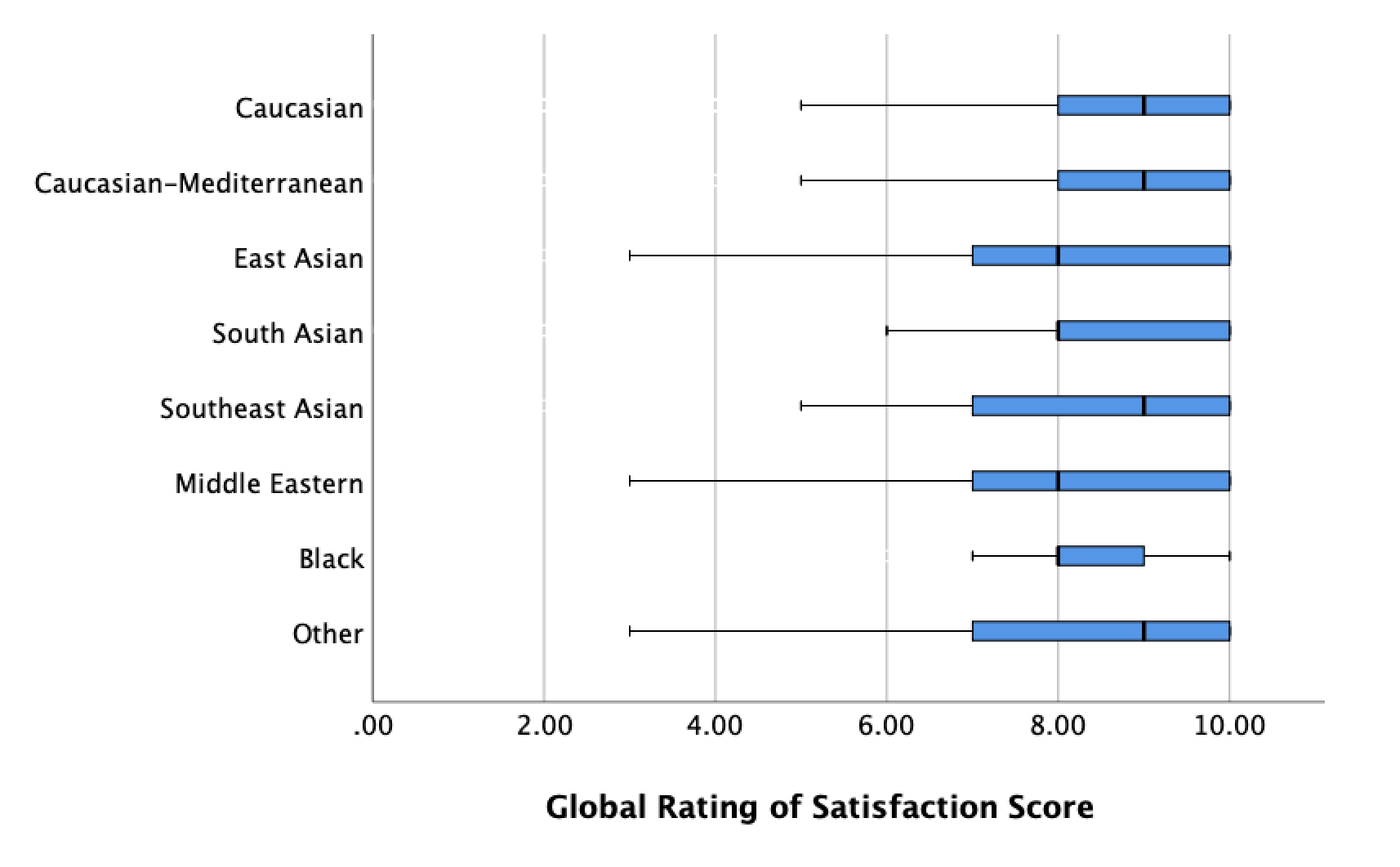
**

**Figure A2 – The Influence of Patient Religiosity on Mean Ratings of Satisfaction by Patient Religion**

^a^ *Error bars represent 95% confidence intervals.*

*^b^ The relative risk ratio could not be calculated for family members of Jewish patients (null value for those who reported being ‘less satisfied’).*

**Table A1 – Multivariable Regression Analysis Evaluating Satisfaction Using a Multi-Categorical Variable Combining Patient Ethnicity and Religion**

| Variables | Multivariable Poisson Regression | |
| --- | --- | --- |
|  | Adjusted Relative Risk (RR) CI (95%) | p-value |
| Patient Ethnicity and Religion  Caucasian/not Muslim vs. Muslim/not Caucasian  Not Caucasian/not Muslim vs. Muslim/not Caucasian | 2.41 (1.17-4.99)  2.17 (0.98-4.79) | 0.017  0.056 |
| Language/Communication Barriers  Usually/always vs. never/sometimes | 0.49 (0.23–1.06) | 0.069 |
| Location of Death  ICU vs. other | 1.51 (1.05–2.19) | 0.028 |

^a^ *Relative risk ratio for Muslim/Caucasian is not presented due to a null value (n=0).*

**Table A2 – High Priority Areas for Improving the Quality of End-of-Life Care**

| **Survey Items** | |
| --- | --- |
| **2** | Language was communication barrier |
| **7** | Doctor listened to concerns |
| **8** | Nurse listened to concerns |
| **9** | Social worker listened to concerns |
| **10** | Chaplain listened to concerns |
| **12** | Kept informed about family members condition |
| **14** | Family member had specific wishes regarding treatment |
| **15** | Discussed Living Will/Power of Attorney with healthcare team |
| **16** | Told how pain would be treated |
| **18** | Received information on medications for pain/shortness of breath |
| **19** | Amount of help dealing with anxiety/sadness |
| **22** | Concerns about personal care needs being met |
| **23** | Wanted more info on what to expect while dying |
| **24** | Amount of support at time of family members death |
| **25** | Amount of support following family members death |
| **28** | Able to access doctor when needed |
| **29** | Doctor spent enough time with you |
| **34** | Clear which doctor was in charge of care |
| **35** | Clear which nurse was in charge of care |

**Table A3 – High Priority Areas for Improving the Quality of End-of-Life Care for the Muslim Patient Population**

| **Survey Items** | |
| --- | --- |
| **4** | Nurse listened to concerns |
| **5** | Chaplain listened to concerns |
| **7** | Kept informed about family members condition |
| **9** | Told how pain would be treated |
| **10** | Amount of help family member got with breathing |
| **11** | Would have liked more information on medications |
| **14** | Concerns about personal care needs being met |
| **16** | Able to access doctor when needed |
| **20** | Satisfied that doctor explained things honestly |
| **21** | Satisfied that you received timely updates |
| **22** | Clear which doctor was in charge of care |
| **24** | Satisfied that health care staff worked as a team |
| **25** | Problems with doctors not knowing medical history |

**Table A4 – Sensitivity Analysis of Cut-Off Values to Measure Satisfaction Among Family Members of Muslim Patients**

| Outcome Measure | Univariable Poisson Regression | |
| --- | --- | --- |
|  | Unadjusted Relative Risk (RR) CI (95%) | p-value |
| Cut-off score ≤3 | 0.39 (0.12-1.18) | 0.095 |
| Cut-off score ≤4 | 0.40 (0.16-1.01) | 0.054 |
| Cut-off score ≤5 | 0.46 (0.22-0.95) | 0.036 |
| Cut-off score ≤6 | 0.67 (0.33-1.37) | 0.273 |
| Cut-off scores ≤3 vs. 4 through 7 vs. ≥8 | 0.58 (0.35-0.97) | 0.036 |
| Ordinal/continuous variable | 0.67 (0.45-0.99) | 0.046 |

## Appendix B

**
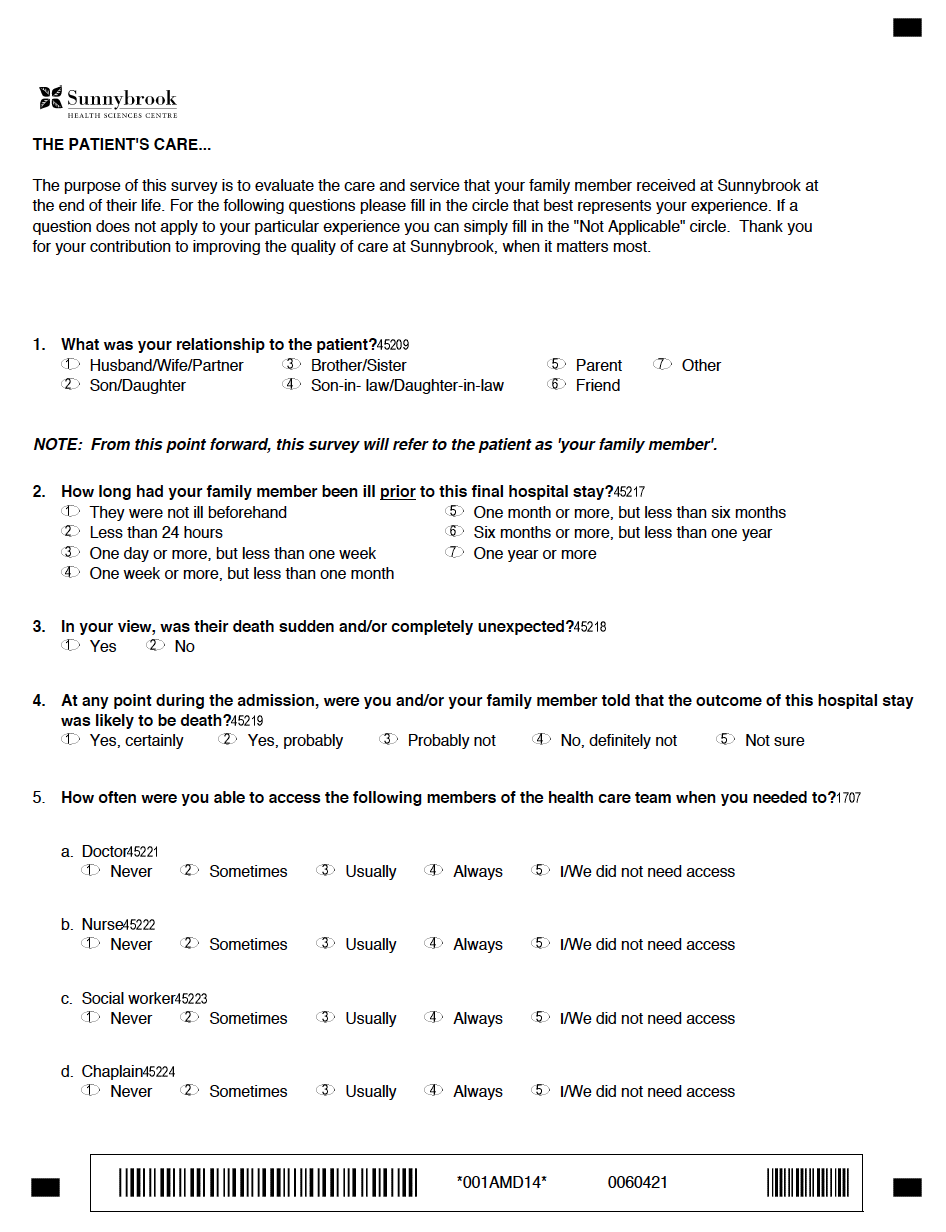
**

**
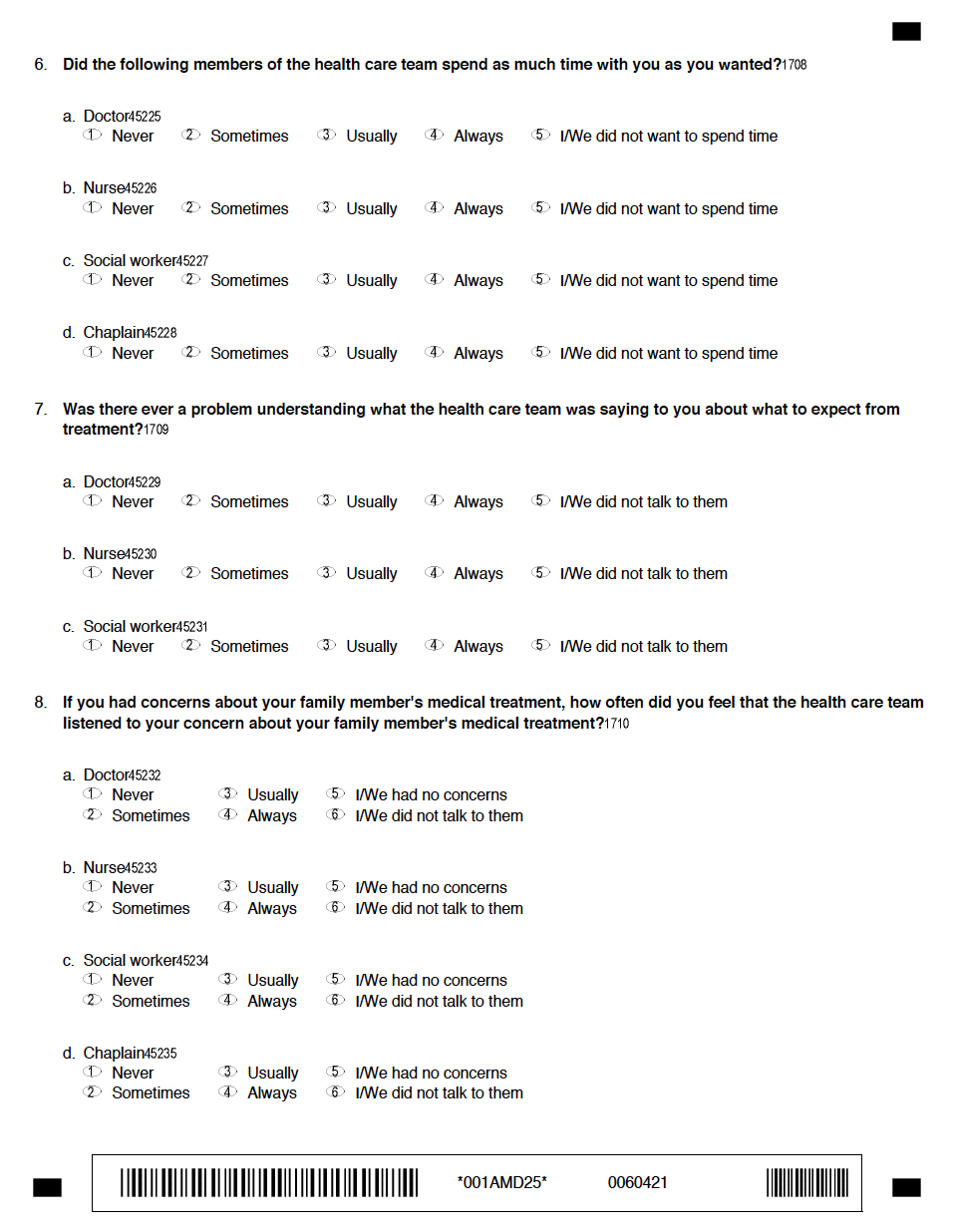
**

##
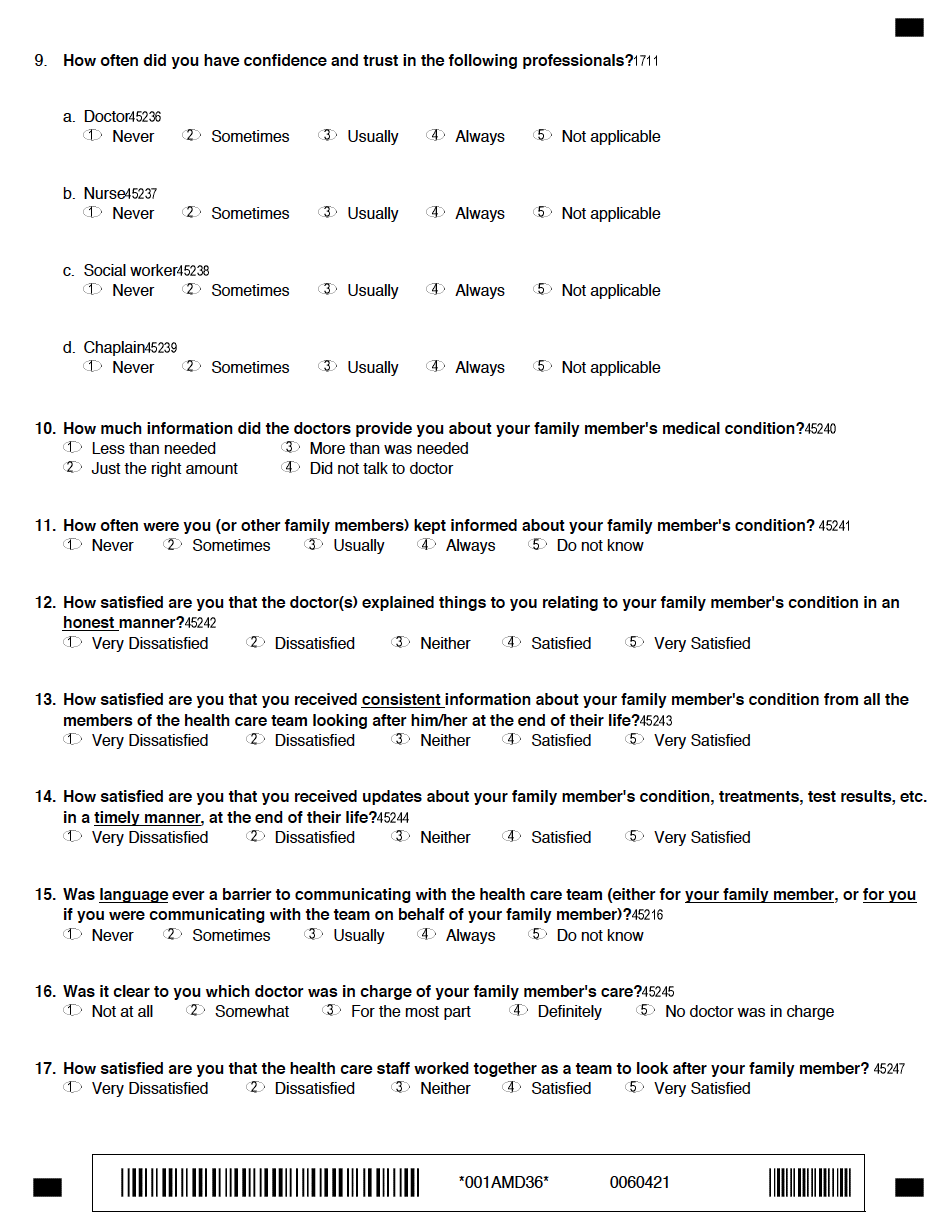


**
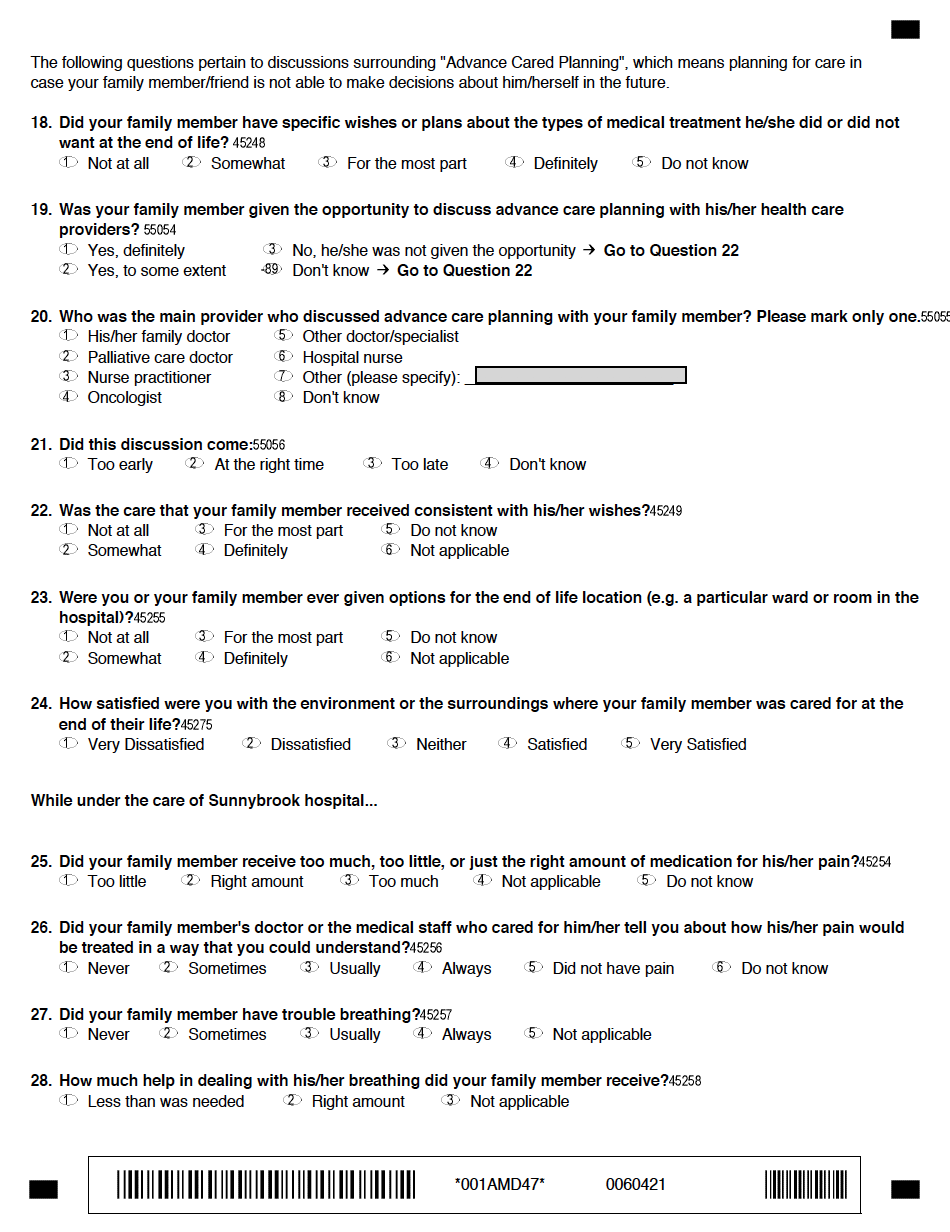
**


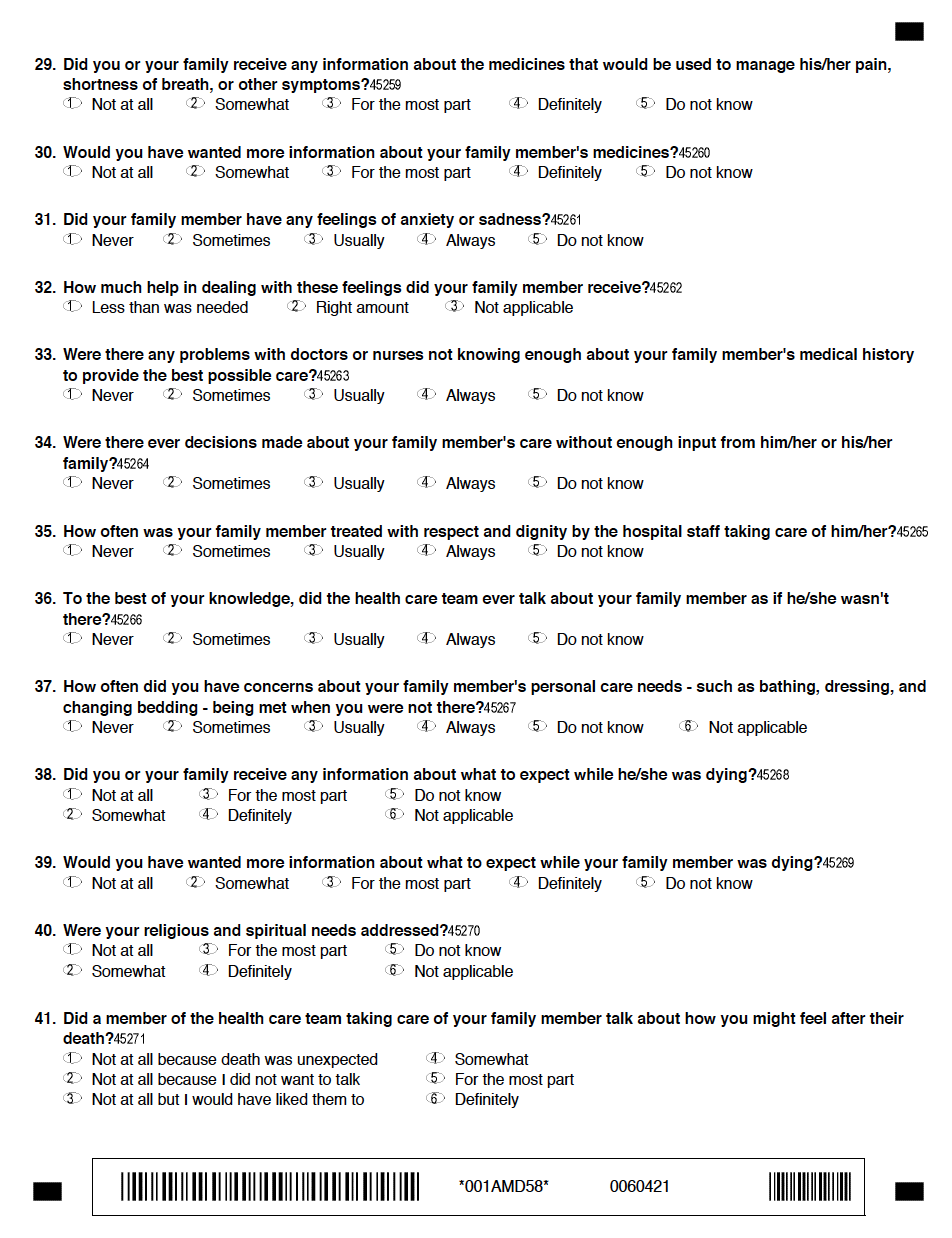


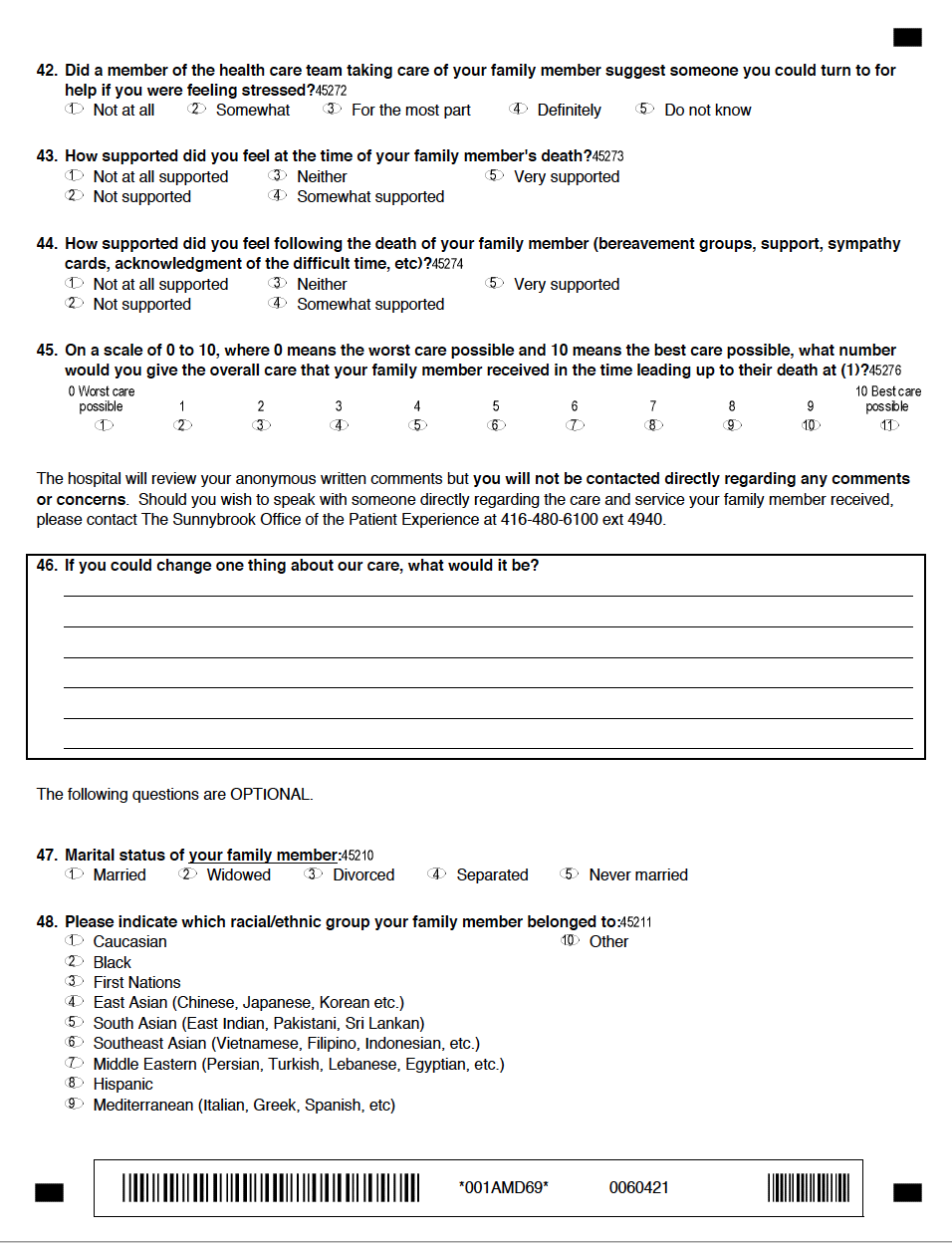


##
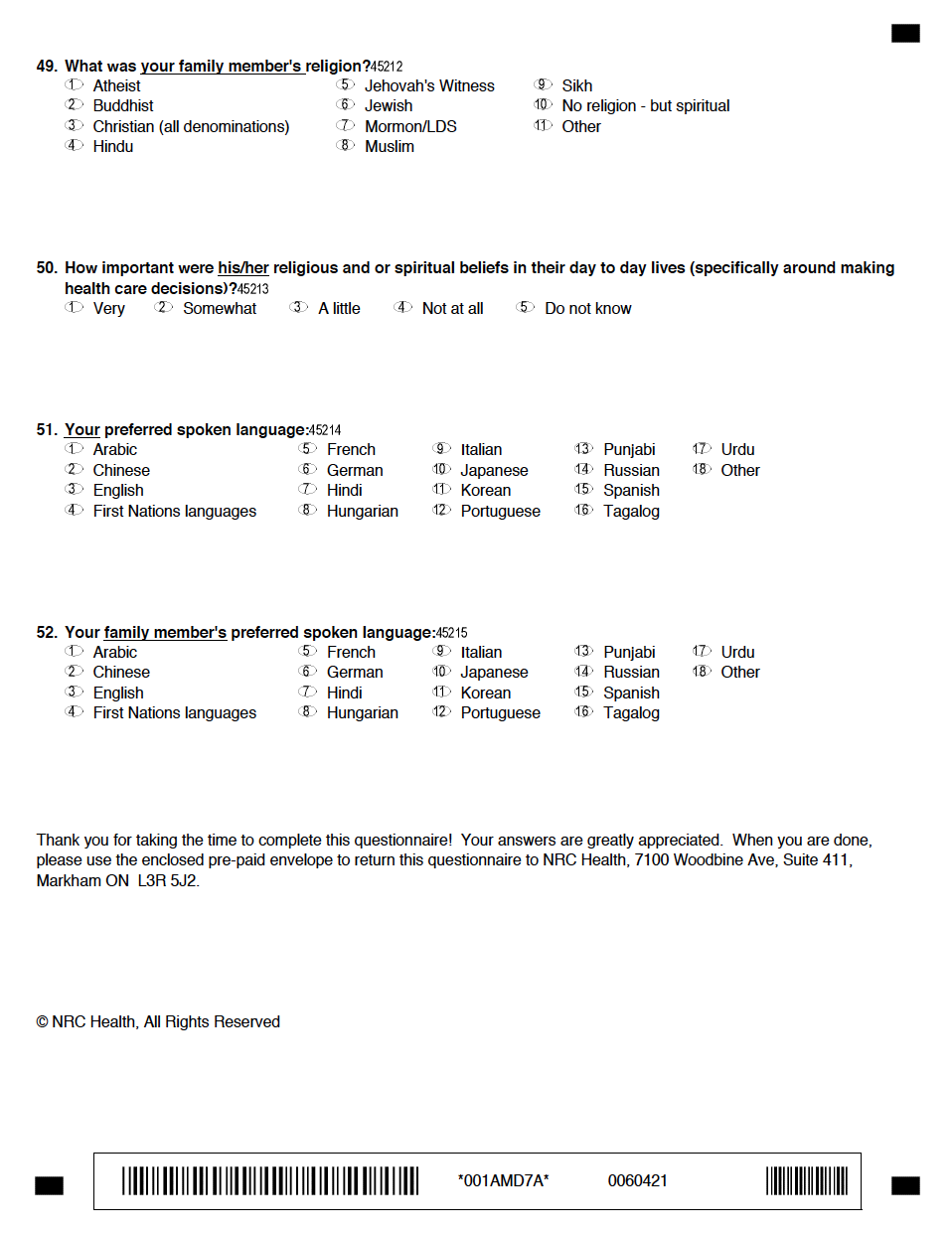


## Appendix C

**Sensitivity Power Analysis (using G*Power):**

**t tests** - Means: Difference between two independent means (two groups)

**Analysis:** Sensitivity: Compute required effect size

**Input:** Tail(s) = Two

α err prob = 0.05

Power (1-β err prob) = 0.8

Sample size group 1 = 1089

Sample size group 2 = 295

**Output:** Noncentrality parameter δ = 2.8035305

Critical t = 1.9616820

Df = 1382

Effect size d = 0.1840129
